# Sex differences in association between body composition and frailty or physical performance in community-dwelling older adults: Erratum

**DOI:** 10.1097/MD.0000000000025059

**Published:** 2021-02-26

In the article, “Sex differences in association between body composition and frailty or physical performance in community-dwelling older adults”,^[1]^ which appeared in Volume 100, Issue 4 of *Medicine*, the rows in Tables 4 and 5 were not correctly aligned. The correct tables are below.

**Table 4:** Univariate and multivariate logistic regression analyses of frailty by body composition.

	Male		Female	
	OR (95% CI)	*P*	OR (95% CI)	*P*
BMI				
Unadjusted	0.926 (0.880–0.975)	***.004***^***∗***^	0.969 (0.922–1.019)	.219
adjusted^†^	0.914 (0.864–0.967)	***.002***^***∗***^	0.973 (0.925–1.023)	.288
BFP				
Unadjusted	1.007 (0.982–1.032)	.600	0.996 (0.973–1.020)	.739
adjusted^†^	0.996 (0.970–1.022)	.757	1.012 (0.986–1.039)	.368
FMI				
Unadjusted	0.983 (0.913–1.060)	.662	0.969 (0.922–1.019)	.219
adjusted^†^	0.952 (0.878–1.033)	.236	1.011 (0.947–1.080)	.745
FFMI				
Unadjusted	0.808 (0.737–0.885)	***.000***^***∗***^	0.969 (0.922–1.019)	.219
adjusted^†^	0.812 (0.736–0.896)	***.000***^***∗***^	0.891 (0.798–0.995)	**.041**^**∗**^
TFMI				
Unadjusted	0.923 (0.826–1.031)	0.157	0.934 (0.847–1.030)	.173
adjusted^†^	0.881 (0.782–0.993)	**0.038**^**∗**^	0.951 (0.857–1.056)	.347

Abbreviations: BFP = body fat percentage, BMI = body mass index, CI = confidence interval, FFMI = fat-free mass index, FMI = fat mass index, OR = odds ratio, TFMI = trunk fat mass index. ^∗^Unless otherwise indicated, the data are reported as relative risk (95% confidence interval). ^†^Adjusted for age, years of education, location of residence, depression, marital status, monthly income, smoking, alcohol drinking, and comorbidities, including diabetes mellitus, dyslipidemia, hypertension, osteoarthritis, and osteoporosis ^∗^*P* < .05

**Table 5:** Univariate and multivariate logistic regression analyses of poor physical performance (SPPB score ≤ 9) and body composition.

	Male		Female	
	OR (95% CI)	*P*	OR (95% CI)	*P*
BMI				
Unadjusted	0.952 (0.894–1.014)	.129	1.046 (1.001–1.093)	***.045***^∗^
adjusted^†^	0.967 (0.903–1.036)	.337	1.058 (1.008–1.111)	***.022***^∗^
BFP				
Unadjusted	1.017 (0.986–1.049)	.293	1.001 (0.978–1.024)	.936
adjusted^†^	1.018 (0.985–1.052)	.277	1.034 (1.008–1.061)	***.010***^∗^
FMI				
Unadjusted	1.113 (0.925–1.113)	.757	1.035 (0.976–1.097)	.245
adjusted^†^	1.028 (0.929–1.137)	.593	1.090 (1.023–1.161)	***.008***^∗^
FFMI				
Unadjusted	0.836 (0.748–0.935)	***.002***^∗^	1.096 (0.996–1.207)	.062
adjusted^†^	0.865 (0.767–0.974)	***.017***^∗^	1.002 (0.899–1.115)	.977
TFMI				
Unadjusted	1.030 (0.898–1.180)	0.674	1.080 (0.984–1.186)	.106
adjusted^†^	1.043 (0.901–1.208)	0.573	1.121 (1.014–1.240)	***.026***^∗^

Abbreviations: BFP = body fat percentage, BMI = body mass index, CI = confidence interval, FFMI = fat-free mass index, FMI = fat mass index, OR = odds ratio, SPPB = short physical performance battery, TFMI = trunk fat mass index. Unless otherwise indicated, data are reported as relative risk (95% confidence interval).

† Adjusted for age, years of education, location of residence, depression, marital status, monthly income, smoking, alcohol drinking, and comorbidities, including diabetes mellitus, dyslipidemia, hypertension, osteoarthritis, and osteoporosis

∗ *P* < .05
